# Improving Electron Extraction Ability and Device Stability of Perovskite Solar Cells Using a Compatible PCBM/AZO Electron Transporting Bilayer

**DOI:** 10.3390/nano8090720

**Published:** 2018-09-12

**Authors:** Hang Dong, Shangzheng Pang, Yi Zhang, Dazheng Chen, Weidong Zhu, He Xi, Jingjing Chang, Jincheng Zhang, Chunfu Zhang, Yue Hao

**Affiliations:** State Key Discipline Laboratory of Wide Band Gap Semiconductor Technology, School of Microelectronics, Xidian University, 2 South Taibai Road, Xi’an 710071, China; donghangxd@163.com (H.D.); 18717344558@163.com (S.P.); dzchen@xidian.edu.cn (D.C.); wdzhu@xidian.edu.cn (W.Z.); hxi@xidian.edu.cn (H.X.); jjingchang@xidian.edu.cn (J.C.); jchzhang@xidian.edu.cn (J.Z.); yhao@xidian.edu.cn (Y.H.)

**Keywords:** perovskite solar cells, aluminum-doped zinc oxide (AZO), electron transporting bilayer, stability

## Abstract

Due to the low temperature fabrication process and reduced hysteresis effect, inverted p-i-n structured perovskite solar cells (PSCs) with the PEDOT:PSS as the hole transporting layer and PCBM as the electron transporting layer have attracted considerable attention. However, the energy barrier at the interface between the PCBM layer and the metal electrode, which is due to an energy level mismatch, limits the electron extraction ability. In this work, an inorganic aluminum-doped zinc oxide (AZO) interlayer is inserted between the PCBM layer and the metal electrode so that electrons can be collected efficiently by the electrode. It is shown that with the help of the PCBM/AZO bilayer, the power conversion efficiency of PSCs is significantly improved, with negligible hysteresis and improved device stability. The UPS measurement shows that the AZO interlayer can effectively decrease the energy offset between PCBM and the metal electrode. The steady state photoluminescence, time-resolved photoluminescence, transient photocurrent, and transient photovoltage measurements show that the PSCs with the AZO interlayer have a longer radiative carrier recombination lifetime and more efficient charge extraction efficiency. Moreover, the introduction of the AZO interlayer could protect the underlying perovskite, and thus, greatly improve device stability.

## 1. Introduction

Despite uncertainties regarding device stability and the usage of lead, metal halide perovskite solar cells (PSCs) have attracted increasing attention from both academia and industry due to their unprecedented properties, such as high absorption coefficients, long charge carrier diffusion lengths, and solution processing approach, since their first use as the active layer for photoelectron chemical cells in 2009 [1,2]. In the past few years, the power conversion efficiency (PCE) of PSCs has been rising dramatically, from 3.8% [3] to present values which are higher than 23% [4]. So far, two main device architectures have been used to fabricate PSCs. One is named the n-i-p structure, which usually involves depositing the perovskite material onto transparent substrates covered with a compact TiO_2_ electron transport layer (ETL) and an optional mesoporous TiO_2_ (or Al_2_O_3_) scaffold layer [5,6]. The other is named the p-i-n structure, which involves depositing the perovskite material onto transparent substrates which are covered with a hole transport layer (HTL), such as the poly(3,4-ethylenedioxythiophene):polystyrene sulfonic acid (PEDOT:PSS) [7,8]. High temperature annealing is usually necessary for the n-i-p structure to attaim high quality TiO_2_ layers compared to the p-i-n structure, which could increase the production cost, and prevent its use in flexible substrates and multi-junction device architectures. As an alternative approach, the p-i-n structure is being used in this paper with PEDOT:PSS as the HTL and [6,6]-phenyl-C61-butyric acid methyl ester (PCBM) as the ETL, due to the low temperature fabrication process and reduced hysteresis effects [7,8].

In order to improve the PCE of p-i-n PSCs, many film growth methods such as the one step method [8], two step sequential deposition method [9,10,11], and the mixed-solvent-vapor annealing method [7] have been developed to achieve a highly uniform, dense, and pin-hole free perovskite films, which can yield more electron-hole pairs upon illumination with light, and thereby reduce the energy loss induced by recombination. With the continuous quality improvement of perovskite films, another issue in regard to interfaces in the PSCs is becoming more and more important, i.e., the p-i-n structure requires that the ETL be deposited on top of the perovskite layer. PCBM as a typical ETL is usually used in this structure. However, if the cathode electrode (Ag, Al) is evaporated onto the PCBM layer directly, there is always an energy barrier at the interface between the PCBM layer and the metal electrode due to the energy level mismatch [12]. Such a PCBM/metal electrode is not optimized for the electron extraction [12]. Therefore, in order to improve the device performance, a method of interface engineering between the PCBM layer and the metal electrode whereby additional layers are inserted has been proposed. In the past few years, some attempts have been proposed to improve the electron extraction properties between the PCBM layer and the metal electrode. For example, by inserting the interlayer of LiF between PCBM and electrode, Seo et al. achieved a PSC with a PCE of 14.1% for a unit cell and 8.7% for the module [13]. By using the polyethylenimine ethoxylated (PEIE) interlayer between the PCBM and electrode, Yang et al. demonstrated a high performance planar heterojunction PSC with a PCE of 14.82% [12]. By inserting the Ca between the PCBM and electrode, Chiang et al. achieved the best PCE, 16.31% [14]. By using the C_60_ and bathocuproine (BCP) interface modified layer, an optimal PCE of 17.9% was obtained [15]. By inserting PFN (poly [(9,9-bis(30-(N,N-dimethylamino)propyl)-2,7-fluorence)-alt-2,7-(9,9-dioct ylfluorene)]) interlayer between the PCBM and electrode, You et al. got an improved PCE of 17.1% [16]. All of these have shown that inserting the interlayer between the PCBM and electrode is an effective way to improve device performance. However, the interlayer materials such as the low workfunction metal (Ca) or organic materials are usually not stable enough. Thus, other interface materials are required. There are many n-type metal oxides such as zinc oxide (ZnO) [17,18], titanium oxide (TiO_x_) [19,20], and tin oxide (SnO_2_) [21,22,23], which has a low work function, and improves the electron extraction ability when used as the interlayer. However, in order to achieve good material quality, these metal oxides usually require a high temperature process, which is not suitable for the p-i-n structure in PSCs because the high process temperature will destroy the underlying perovskite materials. Thus, the means by which a high quality metal oxide may be obtained at a low temperature becomes important for the p-i-n structure in PSCs.

In this work, we adopt a room temperature solution processed Al-doped ZnO (AZO) as the interlayer between the PCBM and Ag electrode to improve device performance. AZO is a wide bandgap material with beneficial properties such as low workfunciton, high electron mobility, high optical transparency, and low-cost [24]. By using the AZO interlayer, the fabricated PSC shows an improved performance with low hysteresis and enhanced device stability. By comparing to devices without the AZO interlayer, it was found that the PSC with the AZO interlayer has a longer radiative carrier recombination lifetime. This may be attributed to the reduction of the energy mismatch between the PCBM layer and Ag electrode by introducing the AZO interlayer, which improved the electron extraction ability. By using the PCBM/AZO bilayer, a PSC with a short current density (Jsc) of 22.82 mA/cm^2^, an open circuit voltage (Voc) of 0.99 V, a fill factor (FF) of 71.68%, and PCE of 16.18% was achieved. Moreover, the AZO interlayer can improve the stability of the device, with the PCE of the best device remaining 86.41% of its initial value after storing the device for over 720 h.

## 2. Materials and Methods

### 2.1. Materials

All solvents and reagents, Aluminum-doped zinc oxide nanoparticle ink (AZO, 2.5 wt.%, Sigma-Aldrich, Saint Louis, MI, USA), Methylammonium iodide (MAI, 99.8%, Dyesol, Queanbeyan, Australia), Formamidinium iodide (FAI, 99.8%, Dyesol, Queanbeyan, Australia), Lead iodide (PbI_2_, 99.999%, Sigma-Aldrich, Saint Louis, MI, USA), Lead chloride (PbCl_2_, 99.999%, Sigma-Aldrich, Saint Louis, MI, USA), Phenyl-C61-butyric acid methyl ester (PCBM, 98%, Nano-c, Westwood, MA, USA), Poly(3,4-ethy-lenedioxythiophene) Poly(styrenesulfonate) (PEDOT: PSS, Clevios PVP Al 4083, Hanau, Germany), N,N’-Dimethylformamide (DMF, 99.8%, Aladdin, Beijing, China), Chlorobenzene (CB, 99.8%, Sigma-Aldrich, Saint Louis, MI, USA), and Isopropanol (IPA, 99.5%, Sigma-Aldrich, Saint Louis, MI, USA), unless stated otherwise, are used as received without further purification.

### 2.2. Film Formation and Device Fabrication

The planer PSCs were fabricated on pre-patterned ITO glass substrates (10 Ω per square, around 2 × 2.5 cm^2^ in size, Zhuhai Kaivo, Zhuhai, China). The patterned ITO glass substrates were sequentially ultrasonic cleaned with 5% decon-90 solution, de-ionized water, acetone, de-ionized water, alcohol at 50 °C for 20 min, respectively. Then the ITO substrates were dried with nitrogen and cleaned in a UV ozone oven for 30 min. A thin layer of PEDOT:PSS was spin-coated on the substrates at 7000 rpm for 45 s, and annealed at 150 °C for 15 min. After that, the substrates were transferred into a nitrogen-filled glovebox. To make a uniform perovskite layer, a perovskite precursor solution consisting of 1.36 M PbI_2_ and 0.24 M PbCl_2_ in the solvent of DMF (named PbX_2_ solution) was stirred for 2 h at 75 °C, and 70 mg MAI and 30 mg FAI were dissolved in the solvent of IPA for the late use. Around 60 μL PbX_2_ precursor solution pre-heated to 75 °C was transferred by pipettes to the PEDOT:PSS covered ITO substrates. Briefly, the spin coating process was programmed to run at 3000 rpm for 45 s. Then MAI and FAI mixed solution was spin-coated on top of the dried PbX_2_ layer at room temperature at 3000 rpm for 45 s. All of the films were thermally annealed on the hotplate at 100 °C for 10 min. Next, a layer of PCBM (20 mg/mL in chlorobenzene) was spin-coated on the top of the perovskite layer at 2000 rpm for 45 s. After that, the AZO solution (8 mg/mL in IPA) was spin-coated on the top of the PCBM layer at 6000 rpm for 45 s, and the thickness of AZO film is about 90 nm measured by Stylus Profiler (Bruker Dektak XT, Bremen, Germany). The devices were finished by thermally evaporated 100 nm Ag. All the devices had an effective area of 7 mm^2^.

### 2.3. Device Characterization

The morphologies of the perovskite layers were measured by scanning electron microscopy (SEM) (JSM-7800F, JEOL Ltd., Tokyo, Japan) and atomic force microscopy (AFM) (Agilent 5500, Santa Clara, CA, USA). X-ray diffraction (XRD) test was conducted on X’ Pert Pro XRD (Bruker Optics, Ettlingen, Germany) and the samples were prepared as the same process of device fabrication. UPS measurements were acquired with a VG ESCA 220i-XL system (VG Instruments, Manchester, UK). The UV source was a He discharge lamp with a photon energy of 21.2 eV. The UV–visible absorption spectra were recorded with an UV–visible spectrophotometer (Perkin-Elmer Lambda 950, Waltham, MA, USA). Photovoltaic performances were measured by using a Keithley 2400 source meter (Tektronix, Inc., Beaverton, OR, USA) under simulated sunlight from XES-70S1 solar simulator (SEN-EI Electric. Co. Ltd, Osaka, Japan) matching the AM 1.5 G standard with an intensity of 100 mW/cm^2^. The system was calibrated against a NREL certified reference solar cell. Incident photo-to-current conversion efficiencies (IPCEs) of PSCs were measured by the solar cell quantum efficiency measurement system (SCS10-X150, Zolix instrument. Co. Ltd, Beijing, China). Transient photocurrent measurement was performed with a system excited by a 532 nm (1000 Hz, 3.2 ns) pulse laser. Transient photovoltage measurement was performed with the same system excited by a 405 nm (50 Hz, 20 ms) pulse laser. A digital oscilloscope (Tektronix, D4105, Beaverton, OR, USA) was used to record the photocurrent or photovoltage decay process with a sampling resistor of 50 Ω or 1 MΩ, respectively. The thickness of AZO film was measured by Stylus Profiler (Bruker Dektak XT, Bremen, Germany). All measurements of the solar cells were performed under ambient atmosphere at room temperature without encapsulation.

## 3. Results and Discussion

**Figure 1:**
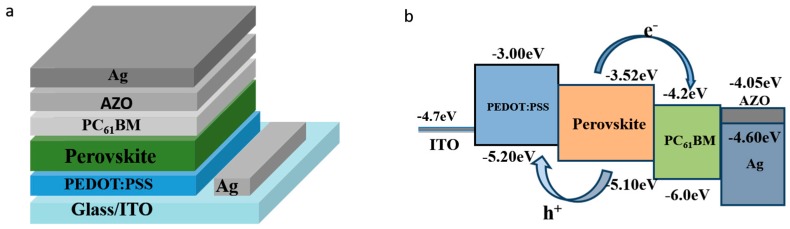
(**a**) Schematic structure of the MA_0.7_FA_0.3_PbI_3-x_Cl_x_ devices in this study: ITO/PEDOT:PSS/ MA_0.7_FA_0.3_PbI_3-x_Cl_x_ /PCBM/AZO/Ag. (**b**) The energy level diagram of the p-i-n solar cell. The energy levels of ITO, the bandgap of PEDOT:PSS, and the bandgap of PCBM were referenced from the previous report [12]. The workfunction of AZO was measured by UPS as stated in the following.

The device structure in this work is shown in Figure 1a, where the PEDOT:PSS and PCBM act as the HTL and ETL, respectively. Here an inverted planar heterojunction structure is used instead of the conventional structure due to its advantages as mentioned earlier. AZO is inserted between the PCBM layer and Ag electrode to decrease the energy mismatch between them, so that the carrier collection may be efficiently improved. Figure 1b shows the schematic band diagram of this work with/without AZO. The energy level mismatch between PCBM and Ag electrode could lead to inefficient carrier transport. One of the methods to enhance the performance of the p-i-n PSCs is interface engineering. It is expected that the AZO interlayer with excellent photoelectric properties can reduce the work function of metal electrode [25,26], so that the carriers can be efficiently collected by the Ag electrode, as shown in Figure 1b.

High quality perovskite material is the key to achieving a high performance PSC. In order to create a uniform and smooth perovskite film, a mixed-solvent-vapor annealing technique is used here, as in the previous report [7]. After spin-coated the MAI and FAI mixed solution, the perovskite films were put on top of a hot plate and covered by a glass petri dish. 40 μL of IPA:DMF (100:1 *v*/*v*) solvent was added in the petri dish around the substrates during the thermal annealing process so that the solvent vapor could react with the perovskite film. Figure S1 reveals the surface morphology of the fabricated perovskite film on the top of the ITO/PEDOT:PSS layer. It is obvious that the perovskite film is uniform and smooth with the grain size approximately 300–500 nm.

**Figure 2:**
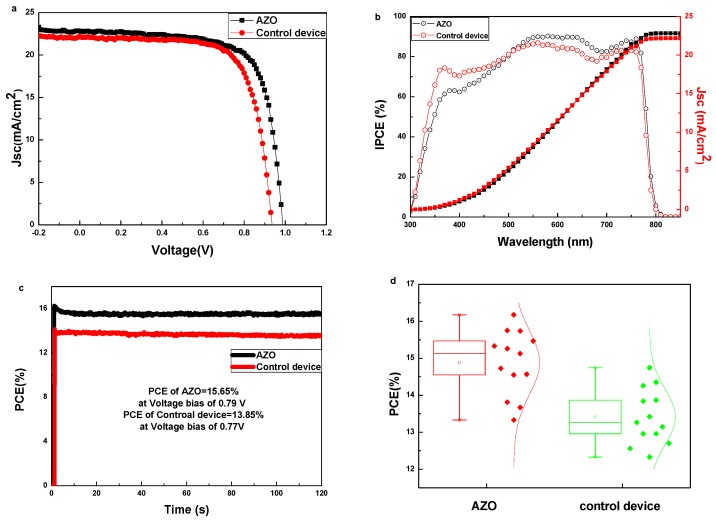
(**a**) Best performed J–V characteristics of PSCs with/without the AZO interlayer measured under 100 mW/cm^2^ AM 1.5G illumination. (**b**) IPCE curves and integrated current density of PSCs with/without the AZO interlayer. (**c**) Steady output characteristics of PSCs with/without the AZO interlayer. (**d**) Statistics result of PCE for PSCs with/without the AZO interlayer.

Figure 2a presents the champion current density-voltage (J-V) curves of devices with/without the AZO interlayer, and Table 1 shows the corresponding device parameters. The champion device with the AZO interlayer achieves a PCE of 16.19%, with a Jsc of 22.82 mA/cm^2^, a Voc of 0.99 V and a FF of 71.68%. Compared with this device, the device without the AZO interlayer gives a PCE of 14.80%, with a Jsc of 22.18 mA/cm^2^, a Voc of 0.94 V and a FF of 71.02%. The device performance with the AZO interlayer is much higher than that without the AZO layer. The higher Jsc, Voc and FF values for the device with the AZO interlayer are supposed to be mainly caused by the suitable work function and efficient charge extraction with the insertion of the AZO layer. Jsc of the devices is also checked by the IPCE spectra, as shown in Figure 2b. It can be seen that the Jsc of the devices integrated from the IPCE spectra matches well with those obtained from J-V measurements. The high IPCE value for the device with the AZO interlayer should be related with the excellent charge transport in the interface of PCBM/AZO bilayer. Figure S2 reveals the J-V curves of PSCs with/without the AZO interlayer measured via both forward and reverse bias sweeps. (Forward scan: from a negative bias −0.2 V to a positive bias 1.1 V, and reverse scan: from a positive bias 1.1 V to a negative bias −0.2 V). It was observed that the PSC with the AZO interlayer shows the neglect photocurrent hysteresis. On the other hand, the PSC without the AZO interlayer shows an obvious photocurrent hysteresis. The hysteresis effect in J-V measurements is one of the most challenging issues for PSCs. It has been shown that it is related to the accumulation of the interface [27]. The neglect photocurrent hysteresis for the device with the AZO interlayer means that there is negligible accumulation at the interface, and thus, better carrier extraction ability. Furthermore, the stabilized PCEs at the maximum power output point were examined, as shown in Figure 2c. The steady-state PCEs are measured to be 15.65% and 13.85% for the devices with/without the AZO interlayer, respectively. They are close to the values obtained from light J-V curves, indicating the reliability of the J-V curve measurement. Both the hysteresis measurement and the stead-state measurement show that the device with the AZO interlayer has the best performance, which is consistent with the PCE measurement shown in Figure 2a. This may be attributed to the fact that with the insertion of the AZO interlayer between the PCBM layer and Ag electrode, a bilayer with excellent charge transport efficiency will be formed.

To confirm the above, the statistic results for the device parameters are summarized in Table S1, Figure 2d and Figure S3a–c. PSCs with the AZO interlayer exhibit a promising average PCE of 14.89% with an average Jsc of 20.99 mA/cm^2^, an average Voc of 0.97 V and an average FF of 73.34%, outperforming the devices without the AZO interlayer, where an average PCE of 13.42% is achieved (the average Jsc, Voc and FF were 22.35 mA/cm^2^, 0.95 V and 63.60% respectively). The parameters of Voc and FF display significant enhancement, indicating the beneficial role of the AZO interlayer with enhanced charge extraction ability.

**Figure 3:**
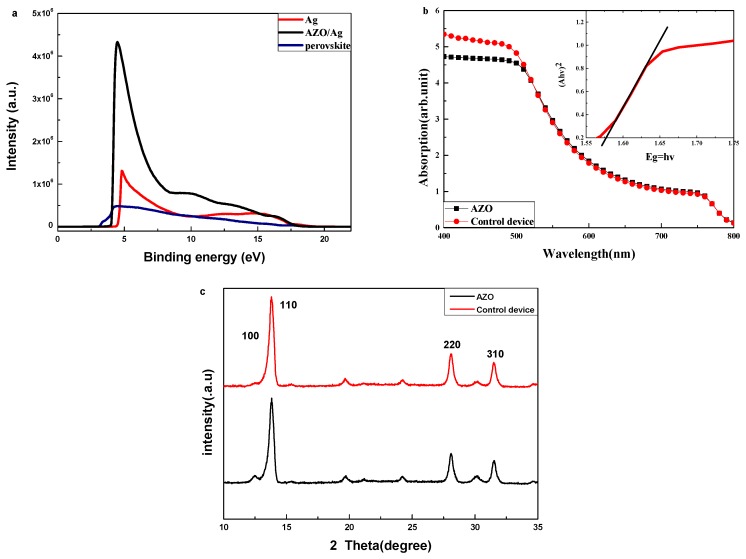
(**a**) UPS measurements of Ag electrode, Ag/AZO and ITO/PEDOT:PSS/perovskite. (**b**) The optical absorption spectra of ITO/PEDOT:PSS/perovskite/PCBM and ITO/PEDOT:PSS/perovskite/PCBM/AZO. inset: the corresponding bandgap of perovskite film. It is noted that the corresponding optical bandgap is about 1.58 eV. (**c**) XRD patterns of ITO/PEDOT:PSS/perovskite/PCBM and ITO/PEDOT:PSS/perovskite/PCBM/AZO.

It is supposed that the PCBM/AZO bilayer could lower the energy barrier between the Ag electrode and the active layer; then, the electron extraction from the active layer to Ag electrode is facilitated. To verify this supposition, UPS measurement was carried out. Figure 3a shows the UPS spectra of Ag electrode, Ag/AZO and ITO/PEDOT:PSS/perovskite to investigate how the energy levels shift. The UPS measurement shows that the work function of Ag is 4.6 eV. After depositing AZO on Ag, there is an obvious shift toward lower work function of 4.05. Figure 3a reveals that the Fermi level (E_F_) of the perovskite film on the ITO/PEDOT:PSS substrate is 3.2 eV, and the energy difference between the valance band maximum and E_F_ is 1.9 eV. We also measured the absorption of ITO/PEDOT:PSS/perovskite/PCBM (as shown in Figure 3b); this indicated that the energy gap of the perovskite is 1.58 eV. Therefore, we can obtain that the conduction band minimum of perovskite film in this work is 3.52 eV. The energy band of PCBM is referenced from the previous report [7] with the conduction band minimum of 4.20 eV and valance band maximum of 6.0 eV. It is obvious that the introduction of the AZO interface layer could decrease the energy level mismatch between PCBM layer and Ag electrode. Thus, electrons produced in the perovskite film could be collected by the Ag electrode more efficiently, which is consistent with the better performance of the device with the AZO interface layer.

Figure 3b shows the UV-vis spectra of ITO/PEDOT:PSS/perovskite/PCBM and ITO/PEDOT:PSS/perovskite/PCBM/AZO. It is obvious that at between 400 and 500 nm wavelengths, the absorbance of the device with AZO is lower than the device without AZO, which matches with the result of IPCE. The result of UV-vis measurement also indicates that the layers of AZO have minimal influence on the light absorption of the perovskite layer between 500 and 800 nm wavelengths. Because the absorbance of AZO layer is very weak and that of ITO/PEDOT:PSS/perovskite/PCBM is very strong, the absorbance of AZO has a little effect on the whole device. The XRD of ITO/PEDOT:PSS/perovskite/PCBM and ITO/PEDOT:PSS/perovskite/PCBM/AZO were also be measured, in order to observe the influence of AZO layer on the perovskite film. As shown in Figure 3c, diffraction peaks at the XRD patterns around 14.17°, 28.52°, and 31.98° are assigned to the (110), (220), and (310) lattice planes of the tetragonal perovskite structure, respectively. The peak at 12.70° is associated with the (100) crystal plane of PbI_2_. The XRD patterns with/without AZO are similar; this demonstrates that the influence of AZO layer to perovskite layer is negligible. Thus, we can conclude that the improved performance of the device with the AZO interlayer is mainly induced by the improved electron extraction ability instead of the improved absorption or crystal quality.

The ITO/PEDOT:PSS/perovskite, ITO/PEDOT:PSS/perovskite/PCBM and ITO/PEDOT:PSS/ perovskite/PCBM/AZO film morphologies and surface textures were also investigated by SEM. It is shown that with the method of mix-solvent-vapor annealing, a high uniform and smooth perovskite film was achieved as shown in Figure S1. Figure S4 shows the top-view SEM images of ITO/PEDOT:PSS/perovskite/PCBM and ITO/PEDOT:PSS/perovskite/PCBM/AZO. It is observed that with the PCBM or PCBM/AZO bilayer deposited on perovskite film their SEM images are breezing. However, the grain size and surface textures are unchanged. This demonstrates that the influence of AZO layer to perovskite layer is negligible, which is consistent with the result of the UV-vis and XRD which we measured above.

**Figure 4:**
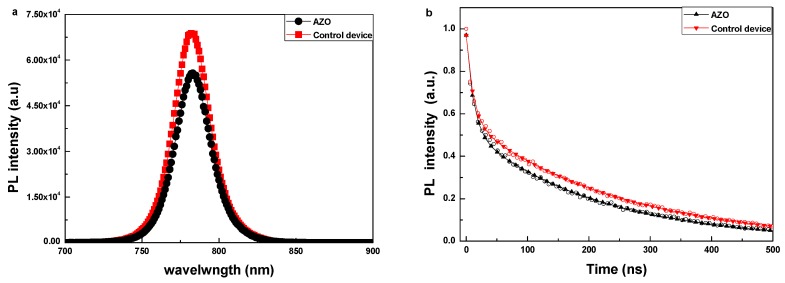

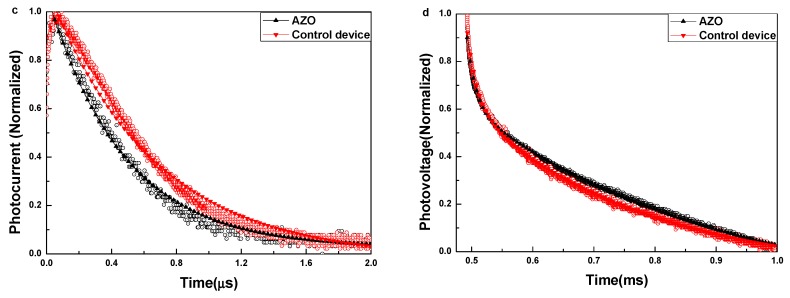
(**a**) Steady-state PL spectra and (**b**) Time-resolved PL spectra of perovskite/PCBM with/without the AZO layer. (**c**) Transient photocurrent and (**d**) transient photovoltage measurements of solar cells with/without the AZO interlayer.

Figure 4a shows the steady-state photoluminescence (PL) spectra of the ITO/PEDOT:PSS/perovskite/PCBM layer with/without the AZO interlayer. The perovskite films were deposited on the glass/ITO/PEDOT:PSS substrates under the same experimental conditions. The excitation light wavelength is 700 nm and the PL emission is collected from the PCBM or PCBM/AZO side. To protect the samples during the measurement, the device was sealed by spin-coating a layer of insulating polymer (PMMA). When the sample has the AZO layer, it could be clearly observed that the peak intensity at 783 nm decreased, which demonstrates that the charge transfer from the perovskite to the ETL is more efficient. This indicates a suppressed non-radiative recombination with the optimized PCBM/AZO bilayer; this is consistent with the obtained UPS results and J-V characteristics discussed above. Figure 4b shows the TRPL behaviors for the sample with/without AZO layer. It is obvious that the sample with the AZO interlayer has a shorter lifespan, and that the sample without the AZO interlayer has a longer one. The lifespan for the sample without the AZO interlayer is around 239.08 ns; this is reduced to 201.84 ns when the AZO interlayer is inserted between the PCBM layer and the Ag electrode. This shows that the photogenerated charge carriers could be efficiently transported to the AZO layer and collected by the Ag electrode. All of these results indicate that efficient electron transfer occurs with the PCBM/AZO bilayer, which is necessary for efficient charge extraction and collection in PSCs, and corresponds with the higher performance and neglect photocurrent hysteresis for devices with the PCBM/AZO bilayer.

The transient photocurrent and photovoltage measurements of PSCs were further performed to verify these results. Figure 4c shows the transient photocurrent of PSCs prepared with/without the AZO interlayer, measured at the short circuit condition. It was found that the device with the AZO interlayer has faster extraction (467.58 ns) than the device without the AZO interlayer (648.25 ns), indicating that the device former possesses much more efficient charge extraction and charge transport properties. Hence, the Voc and FF are enhanced. The transient photovoltage is used to determine the charge recombination lifetime, as shown in Figure 4d. The charge recombination lifetime of the device with the AZO interlayer increases to 0.41 ms, compared to that without the AZO interlayer (0.27 ms), indicating that the charge recombination is efficiently suppressed with the introduction of the AZO interlayer. These transient photocurrent and photovoltage measurement results are consistent with the PL measurement results.

**Figure 5:**
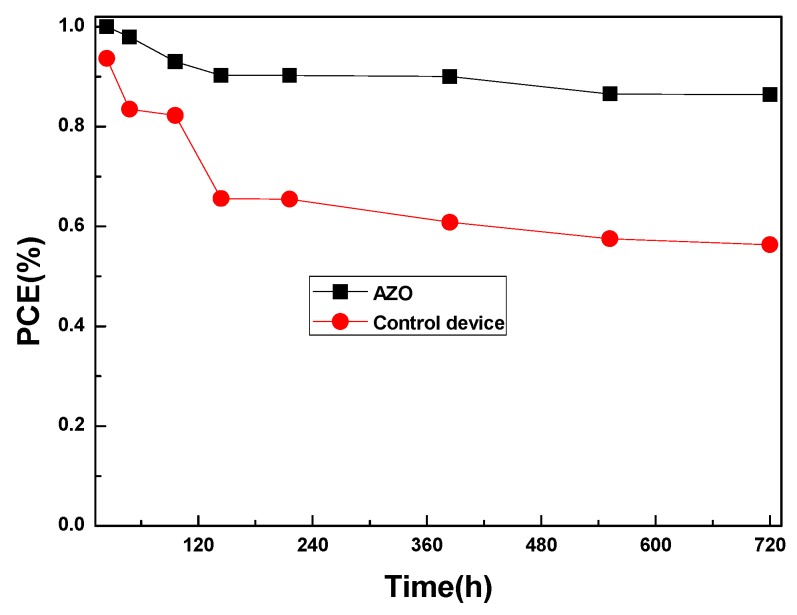
Stability of unencapsulated PSCs with/without the AZO interlayer.

Finally, we studied the stability of the device by storing it for 720 h. As shown in Figure 5 and Figure S5a–c, the device with the AZO interlayer exhibits remarkable PCE stability, retaining 86.41% of its initial value even after storing for over 720 h. The Jsc remained almost unchanged, with a slightly decreased FF value. The Voc has a small increase in the first 100 h, and stays around 1.00 V in the following measurement, while for the device without the AZO interlayer, the PCE decreases quickly to 60.14% of its initial value for the same storage condition and duration, which is mainly caused by the decreased FF and Jsc. These results suggest that the PSC with the PCBM/AZO bilayer exhibits better stability.

## 4. Conclusions

In conclusion, we have demonstrated a stable and efficient p-i-n structure PSC with a PCBM/AZO electron transporting bilayer. The insertion of the AZO interlayer could greatly improve the performance of PSCs; the best-performing device exhibits a PCE of 16.18% with a Jsc of 22.82 mA/cm^2^, a Voc of 0.99 V, and a FF 71.68%. The UPS measurement shows that the AZO interlayer can decrease the energy offset between PCBM and metal electrode. The measurements of PL, TRPL, transient photocurrent, and transient photovoltage show that the PSC with the AZO interlayer has a longer radiative carrier recombination lifetime, and more efficient charge extraction efficiency. In addition, the PSC with the AZO interlayer shows an improved stability.

## Figures and Tables

**Table 1:** Photovoltaic performances of PSCs under AM 1.5G illumination (100 mW/cm^2^).

	Jsc (mA/cm^2^)	Voc (V)	FF (%)	PCE (%)
AZO	22.82	0.99	71.68	16.19
Control device	22.18	0.94	71.02	14.75

## Supplementary Materials

The following are available online at http://www.mdpi.com/2079-4991/8/9/720/s1, Figure S1: Top-view SEM image of the surface morphology of perovskite, Figure S2: (a) J–V curves in forward and reverse scans of PSCs with AZO, (b) J–V curves in forward and reverse scans of PSCs without AZO, Figure S3: Statistics results of Jsc (a) Voc (b) and FF (c) for PSCs with/without AZO, Figure S4: SEM graphs of (a) ITO/PEDOT:PSS/perovskite/PCBM and (b) ITO/PEDOT:PSS/perovskite/PCBM/AZO, Figure S5: Stability of (a) Jsc (b) Voc and (c) FF for unencapsulated devices with/without AZO, Table S1: Statistical results of perovskite solar cells under AM 1.5G illumination (100 mW/cm^2^). The standard deviation results are derived from 12 perovskite solar cells.
