# Supplementary material for: Improving Electron Extraction Ability and Device Stability of Perovskite Solar Cells Using a Compatible PCBM/AZO Electron Transporting Bilayer
Source: Nanomaterials (Basel). 2018 Sep 12;8(9):720. doi: 10.3390/nano8090720 (PMC6164653; doi:10.3390/nano8090720)
Supplement: Supplementary file 1 [file nanomaterials-08-00720-s001.zip › nanomaterials-345462-supplementary.pdf]

Supplementary Information

Improving Electron Extraction Ability and Device Stability of Perovskite Solar Cells by Using a Compatible PCBM/AZO Electron Transporting Bilayer

Hang Dong, Shangzheng Pang, Yi Zhang *, Dazheng Chen, Weidong Zhu, He Xi, Jingjing Chang, Jincheng Zhang, Chunfu Zhang * and Yue Hao

State Key Discipline Laboratory of Wide Band Gap Semiconductor Technology, School of Microelectronics, Xidian University, 2 South Taibai Road, Xi'an 710071, China; donghangxd@163.com (H.D.); shangzhengpang@qq.com (S.P.); dzchen@xidian.edu.cn (D.C.); wdzhu@xidian.edu.cn (W.Z.); hxi@xidian.edu.cn (H.X.); jjingchang@xidian.edu.cn (J.C.); jchzhang@xidian.edu.cn (J.Z.); yhao@xidian.edu.cn (Y.H.)

* Correspondence: hyper_sys@163.com (Y.Z.); cfzhang@xidian.edu.cn (C.Z.)

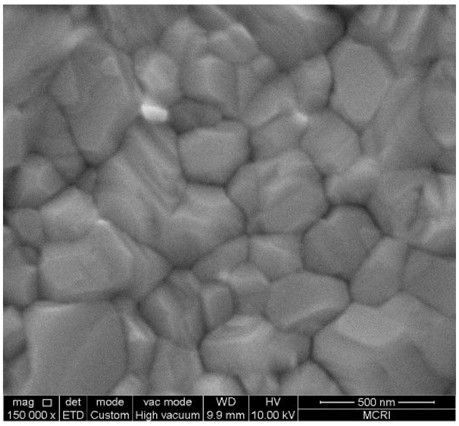

Figure S1. Top-view SEM image of the surface morphology of perovskite.

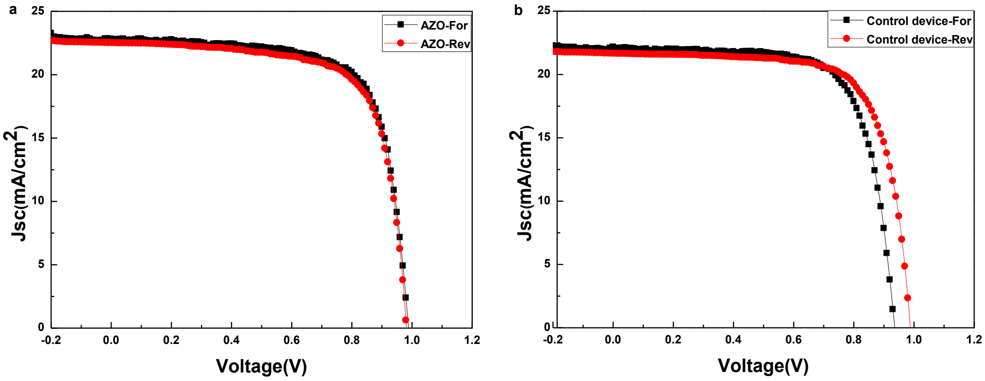

Figure S2. (**a**) J–V curves in forward and reverse scans of PSCs with AZO, (**b**) J–V curves in forward and reverse scans of PSCs without AZO.

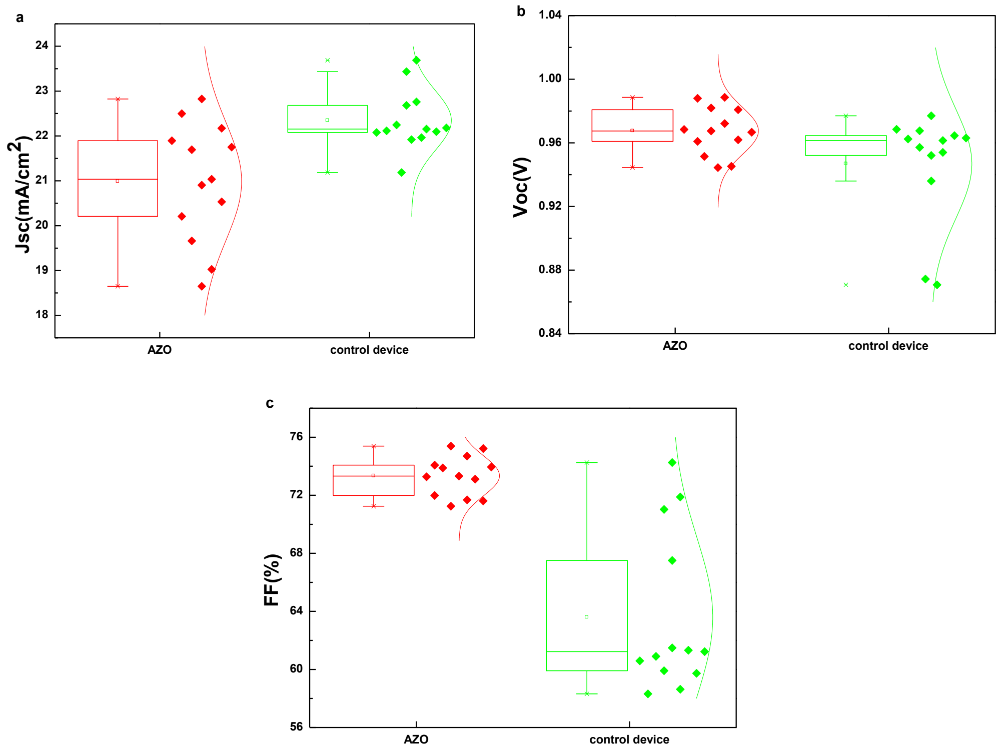

Figure S3. Statistics results of Jsc (**a**) Voc (**b**) and FF (**c**) for PSCs with/without AZO.

Table S1. Statistical results of perovskite solar cells under AM 1.5G illumination (100 mW/cm^2). The standard deviation results are derived from 12 perovskite solar cells.

	J_{sc} (mA/cm^2)	V_{oc} (V)	FF (%)	PCE (%)
AZO	20.99 ± 1.32	0.97 ± 0.014	73.34 ± 1.3	14.89 ± 0.84 (16.19)
Control device	22.35 ± 0.66	0.95 ± 0.033	63.60 ± 5.3	13.42 ± 0.72 (14.75)

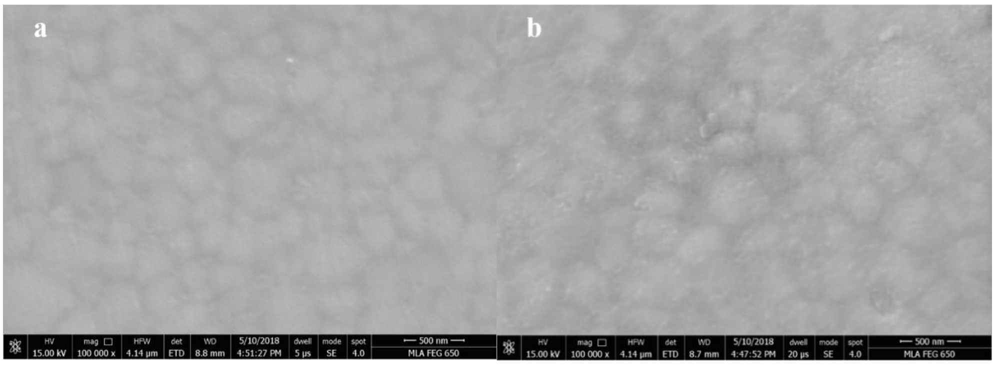

Figure S4. SEM graphs of (**a**) ITO/PEDOT:PSS/perovskite/PCBM and (**b**) ITO/PEDOT:PSS/perovskite/PCBM/AZO.

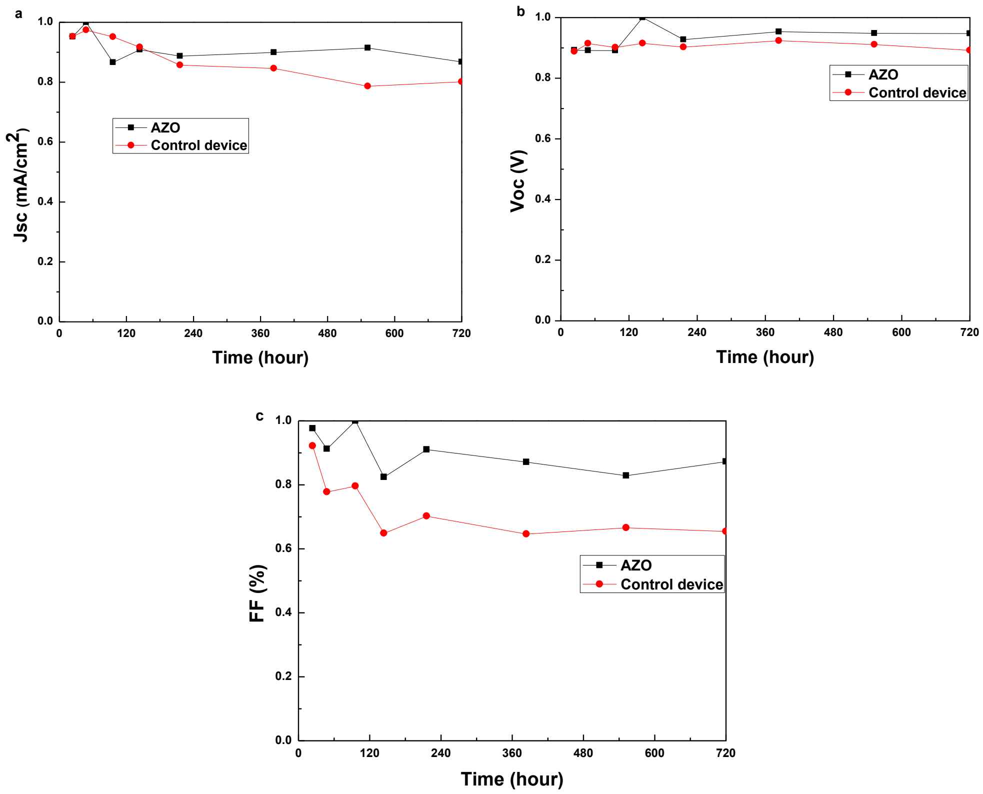

Figure S5. Stability of (**a**) Jsc (**b**) Voc and (**c**) FF for unencapsulated devices with/without AZO.
